# Europe, public debts, and safe assets: the scope for a European Debt Agency

**DOI:** 10.1007/s40888-021-00236-6

**Published:** 2021-07-18

**Authors:** Massimo Amato, Everardo Belloni, Paolo Falbo, Lucio Gobbi

**Affiliations:** 1 Bocconi University, Milan, Italy; 2 Politecnico di Milano SoM, Milan, Italy; 3 University of Brescia, Brescia, Italy; 4 University of Trento, Trento, Italy

**Keywords:** European Debt Agency, Safe asset, Eurobonds, Public debt, Perpetual loans, G2, G28, H63, H81

## Abstract

The Covid-19 crisis has radically changed the game for world and EU-economies, and urged for a reappraisal of the guidelines for a healthy management of public expenditure. This requires a deep rethinking of the role of public debt in modern capitalistic economies and of efficient, equitable and politically viable ways of financing it. This paper outlines the main operating framework of a Debt Agency tasked with the management of the Eurozone sovereign debts and the creation of a truly European safe asset. The framework leverages on the potential irredeemable nature of sovereign debts in order to build a common bond. By structurally filtering liquidity risk, the Debt Agency can price the Member States’ funding costs by referring only to their credit risk, as defined by EU agreed rules. The common bond issued by the Debt Agency thus avoids mutualisation by design; hence, it can be directly bought by the ECB. Due to its structural intertemporal sustainability, the Debt Agency’s framework delineated in this paper can serve as a benchmark for institutional and political decisions. In this perspective, a counterfactual exercise has been conducted in order to evaluate the future potential impact of the Debt Agency as well as the past distortions in market pricing of Member States’ fundamental risk due to market mispricing of the liquidity risk.

## Introduction: Eurozone debts and Eurobonds

The Covid-19 emergency has proved to be a watershed in how the issue of fiscal policy and its financing is addressed (Baldwin & Di Mauro, 2020; Carnazza & Liberati, 2021; Draghi, 2020; ECB, 2020a, b; Galí, 2020; Kapoor & Buiter, 2020; Krugman, 2020; Morelli & Seghezza, 2021; Romer & Garber, 2020; Stiglitz, 2020). Perhaps it is not only a watershed but also a point of no return.

At the Eurozone level, the problems of insufficient performance on the demand side, which were clearly apparent even before the Covid-19 crisis, have been joined by problems of inefficiency on the supply side, hence by problems of coordination between demand and supply. Moreover, on considering the economic effects of the prolonged duration of the pandemic and the level of public expenditure planned in the USA, it cannot be excluded that the fiscal expansions provided until now in Europe could prove insufficient.

What is at stake in this new Covid and post-Covid scenario is twofold. On the one hand, the gaps opened by the fall in income due to repeated waves and subsequent lockdowns must be filled. On the other hand, filling those gaps cannot always leverage on re-establishment of the ‘old normal’: the issue emerges of the investments required by a deep re-infrastructuring of the EU economy, since the restart of production to close the gaps will entail structural changes as well as redefinition of the relationships among sectors (Baldwin & Evenett, 2020; Bamber et al., 2020; Fornaro & Wolf, 2020; Gereffi, 2020).

The Covid crisis has been generated by a symmetrical and completely exogenous shock; but it has had, and will continue to have, largely asymmetrical effects. This is particularly relevant in the EU and in the Eurozone, where integration mechanisms have never included the common financing of public debts in the name of a strict fiscal policy discipline and of a rigid notion of convergence (see Table 1).

**Table 1:** Effects on public debts of Covid-19 crisis. Source: ECB 2019

Country	Public debt 31/12/2019	Public debt 31/12/2020	D2021/Pd2020 (%)
Austria	280,340	315,160	12.42
Belgium	467,172	514,965	10.23
Cyprus	20,958	24,829	18.47
Estonia	2372	4953	108.78
Finland	142,874	164,266	14.97
France	2,379,503	2,650,116	11.37
Germany	2,057,627	2,325,463	13.02
Greece	331,073	341,023	3.01
Ireland	204,223	218,158	6.82
Italy	2,409,942	2,573,386	6.78
Latvia	11,247	12,750	13.37
Lithuania	17,524	23,061	31.59
Luxembourg	13,978	15,942	14.05
Malta	5703	6960	22.05
Netherlands	394,670	434,931	10.20
Portugal	249,977	270,492	8.21
Slovakia	45,275	55,181	21.88
Slovenia	31,744	37,429	17.91
Spain	1,188,820	1,345,570	13.19

Since the inception of the euro, this has meant the exclusion of any mutualisation and transfer mechanism intended to absorb asymmetric shocks.

The exceptional nature of the present situation and its likelihood of being a point of no return have highlighted the need for new ways to finance a fiscal manoeuvre unprecedented in scale and scope.

Hence policy views that were unthinkable before the emergency became commonly shared. In countries in which the circuit with the central bank and treasury is already in operation, the shift to the ‘new normal’ has been smoother. For the Eurozone, it has implied more dramatic changes: in short, suspension of the ‘old normal’.

Undoubtedly, the debate on public debt and its financing, as well as on the role that sovereign debts can play within economies and financial markets, has begun to be revisited. Once again, the shift has been more dramatic in the EU context, where the previous public debt crisis was tackled with few and only temporary deviations from the procedures dictated by financial orthodoxy. Nowadays, orthodox approach is considered insufficient and dangerous by many economists (Baldwin & Di Mauro, 2020; Blanchard & Brancaccio, 2019; Blanchard & Leigh, 2013; Cerniglia & Saraceno, 2020; De Grauwe, 2013; Fatás & Summers, 2018).

It is precisely those deviations, their amplification and their ‘institutionalization’ that are now at stake. We are in a situation in which some radical decisions, taken first by the ECB, and then also by the European Commission (EC), seem to evidence not only the technical feasibility of a reform, but also the political will to change some central aspects of political and monetary union.

In this perspective, an institution intended to efficiently perform the common management of the Eurozone debt would prove not only fundamental for the future of the Eurozone, but also, as we shall show, would have proved fundamental since its inception.

Let us start with a quick update on the situation.

The European response to the Covid-19 shock has taken various forms, but in all cases, it has shown an increasingly decisive orientation of the EU institutions (in particular the Commission, but also the ECB) towards a progressive extension of the scale and scope of the instruments put in place.

As regards the European Commission, the process began with suspension of the Stability and Growth Pact and an initial aid package of around 540 billion euros. It comprised: (i) the possibility for Member States to obtain loans from the European Stability Mechanism for a maximum amount of about 2% GDP in 2019, with light conditionality, for health-related expenditure; (ii) enhancement of the ability of the European Investment Bank to grant loans to businesses of up to 200 billion euros; and (iii) the activation of the SURE (Support to mitigate Unemployment Risks in an Emergency) programme for 100 billion euros.

Concerning the financing of these new tools, a debate has arisen in which some economists and practitioners have proposed the issue of sovereign perpetuities (Giavazzi & Tabellini, 2020; Soros, 2020). In the wake of this debate, proposals have also been made by Member States, like the Spanish one, for instance (Reuters, 2020). Eventually, the European Council formulated a proposal to establish a Recovery Fund (also called NextGenerationEU) of 750 billion euros. After the negotiations in July 2020, the fund provided for the disbursement of 390 billion euros in subsidies and 360 billion euros in loans. Subsequently, specific programmes under the long-term budget for 2021–2027 were reinforced by 15 billion euros.

Even if we are already in the phase in which the money will begin to be disbursed, the Recovery Fund, notwithstanding two important innovations, still involves a ‘great unknown’.

The innovations are (i) that it will be directly financed by the markets through the issue of common bonds directly backed by the EU budget; and (ii) that it will involve some kind of mutualisation, since the disbursements of subsidies to individual countries will not respect their contribution to the establishment of the fund and to the European budget.

The ‘great unknown’ is the temporary vs. definitive nature of the operations envisaged by the fund, and therefore the temporary vs. definitive nature of the instruments provided to finance it. What we know for now is that the common bonds issued to finance the NGEU will have different maturities, and that the commitment is to repay them in full by 2058, with instalments starting from 2028 (ECB, 2020c). In this regard, the European Gordian knot is still untied.

Despite this uncertainty, the demand expressed by the markets for those common bonds has been very high since the first issuances (EC, 2020). This is proof that the potential demand for European safe assets is very high, and that these common bonds are already considered as European safe assets. Therefore, there are good economic and institutional reasons to methodically approach the question of the possible rollover of the common debt, and to provide innovative operational solutions.

Issues relating to the safe asset, with or without mutualization, and its potential rollover, are also closely linked to considerations concerning the possible synergy between fiscal and monetary policy. Because the expansions of public deficits required by a pandemic will not be reabsorbed quickly, the expansion of public spending will probably become a permanent element of the post Covid-19 scenario. Hence, the problem arises of optimizing the management of fiscal spaces at the level of the entire EU and of the Eurozone.

This scenario raises the issue of mutualisation and of moral hazard, at least as regards fiscal policy within a monetary union (Arnold et al., 2018; Batini et al., 2020; Beetsma et al., 2018; Berger et al., 2019). This is crucial because, depending on how it is resolved, the issue will affect the overall expansion of the fiscal space in the EU.

Furthermore, if the issuing of common bonds were to become permanent and were financed with common tax revenues, a problem would arise concerning the European debt ratio with respect to outstanding and future national debts.

As regards monetary policy, given the growing amount of cumulated public debts, it is likely that the ECB will stably assume an active role, with all that this entails in terms of the interpretation of its statutory mandate. To date, the ECB has strengthened the targeted long-term refinancing operations (TLTRO III) and activated targeted long-term refinancing operations linked to the pandemic emergency (PELTRO) at subsidized rates.^1^ As for the Pandemic Emergency Purchase Programme (PEPP), the ECB will continue its purchases with a total envelope of € 1,850 billion. Here too an important novelty emerges: unlike the PSPP, the PEPP involves an abandonment of the capital key rule. This abandonment was declared non-permanent, but at the same time it proved to be a necessary condition for the governance of spreads on financial markets. Also in this case, therefore, there is a problem concerning future orientations. Depending on how the issue is resolved, the potential of monetary policy and the conditions of debt sustainability for Member States, and indirectly also for the common debt, will change (ECB, 2020a, 2021).

In any case, the mix of fiscal expansion (increased debts) recorded to date, combined with the monetary support for its financing (ECB role) highlights a structural problem that gripped the Eurozone even before the pandemic, and especially after the sovereign debt crisis: that is, the problem of a European safe asset (a “safe Eurobond”) able to support the stability of financial markets in times of turmoil (Beck et al., 2011; Bonnevay, 2010; Bruegel, 2020; Brunnermeier et al., 2011, 2017; De Grauwe & Moesen, 2009; Delpla & von Weizsacker, 2010, 2011; Dosi et al., 2018; Giudice et al., 2019; Gros & Micossi, 2009; Hellwig & Philippon, 2011; Juncker & Tremonti, 2010; Leandro & Zettelmeyer, 2018a; Monti, 2010; Ubide, 2015).^2^

At stake are the nature of both common bonds and the issuer thereof, as well as the structural conditions of their financeability.

Some important steps have already been taken in the direction of new tools and perspectives for EU integration, so that one may speak of “tools of a surrogated federalism” (Saraceno, 2020). However, as far as public debt is concerned, a structural solution continues to be needed. This solution must deal not only with the possible issue of common European bonds, but also with the common management of the national debts accumulated so far, since they will not decrease for a long time yet, alongside the likely growing quota of common debt.

Absent an outright federalist solution, the institution that could take on this task, issuing in return a full-fledged European safe asset, could be what we call here a ‘European Debt Agency’. As we shall show in more depth later, this project is compatible with many of the proposals that are under the policymakers’ scrutiny. In particular, it will be able to take over from them in the medium term by absorbing any one-off issue of common bonds.

By sketching the essential features of a new institution, our paper intends to address all these issues, offering a structural solution and a methodological perspective for European integration on a collaborative, not competitive, basis.

The paper is divided into five sections. The foregoing Sect. 1 has introduced the topic. The Sect. 2 briefly surveys the literature on safe assets and Eurobonds. The Sect. 3 describes our proposal in its institutional and technical details. The Sect. 4 deals with simulations, while the Sect. 5 is devoted to conclusions.

## Literature review

In this paper we adopt the broad and formal definition of a safe asset proposed by Caballero et al. (2017): “a safe asset is a simple debt instrument that is expected to preserve its value during adverse systemic events”.

In the above definition, three components are of key importance: (i) the relative simplicity of the instrument; (ii) the expected stability of its long-term value; (iii) its resilience to all sorts of events, also and especially systemic ones. For an asset to be safe, all three of these factors need to be present. Inversely, the lack of only one of them jeopardizes its safety.

The demand for safe assets depends on the prominent role that this asset class plays in the everyday operations of international financial markets (Golec & Perotti, 2017 for a review). Importantly, safe assets are used by banks and various financial institutions as high-quality (high-liquidity) collaterals in transactions. Furthermore, safe assets are widely used by central banks for both conventional and unconventional monetary policies, and by investment funds as a benchmark to price riskier assets as well as a store of value.

Conversely, a safe asset shortage has significant macroeconomic (Caballero & Farhi, 2015) as well as financial implications: a shortage of safe assets as collateral constrains firms’ borrowing capacity (Gorton & Ordonez, 2014). This can trigger a recession phase or act as a financial accelerator that amplifies a shock that has hit an economy (Panetta et al., 2009). On the other hand, a safe asset shortage could encourage governments to increase their public debts in order to fill the gap. This is the case as long as the cost of public debt is lower than the rate of growth of the issuer’s country (Blanchard & Summers, 2017).

The problem of the shortage of safe assets is particularly marked in Europe. And it has been so even since before the Covid-19 crisis (see Table 2).

**Table 2:** Eurozone Public Debts, source ECB 2021

Rating 31/12/2020	Outstanding debt	Proportion (%)
AAA	2,776,336	24.49
AA	3,649,460	32.20
A	1,699,107	14.99
BBB	2,868,706	25.31
BB	341,023	3.01
Total	11,334,631	100.00

Indeed, from the establishment of the European Monetary Union (EMU) until the European sovereign debt crisis, most of the bonds from Eurozone countries enjoyed a safe asset status that many of them lost during the 2010 financial turmoil. This story shows that the resilience of an asset, and hence its safety, can be affected by events. This is what we will show in the counterfactual analysis proposed in Sect. 4: the GFC meant that many Member States’ debts in the Eurozone lost their status as safe assets, and strengthened the safe asset status of others through a ‘flight to quality’. However, since it is questionable that the systemic adverse event of a shutdown of the euro could preserve the safe asset status even for the strongest Member States, the question arises: what about a common European safe asset? Should it not be safer than any single national public debt bond?

The answer is undoubtedly ‘yes’. What the Eurozone has clearly needed since the 2010 turmoil, and will need even more strongly after the Covid-19 emergency, is a common safe asset able to structurally reabsorb the current divergence in yields among Eurozone Member States’ sovereign bonds.

The shortage of safe assets in the Eurozone and the lack of a true European bond are two sides of the same problem. The question emerged in all its clarity in the early stages of the European sovereign crisis, when the spreads between the yields of public debts began to widen due to a sudden stop of capital inflows. This halt caused a flight to quality to Northern countries’ sovereign debts, and a ‘flight from liquidity risk’ which triggered self-fulfilling expectations that eventually led, in the case of the downgraded Member States, to an overshooting of their market yields (see Paniagua et al., 2017; Merler and Pisani-Ferry 2012, as well as Sect. 4 below).

The widening of spreads could not be solved in the current framework of the Eurozone with proactive action by the ECB, since the latter cannot act on the markets outside the capital key clause except in short periods of derogation. The ECB was effective in stopping the absolute growth of certain spreads, but ineffective in reabsorbing the divergence among Member States’ bonds yields, precisely because of the capital key. And this was no accident: in the absence of a Eurozone Treasury entitled to issue fully mutualized bonds analogous to US T-bills and T-bonds, the ECB could not but follow the capital key rule. This led to the appearance of negative yield on AAA bonds (see Sect. 4 below).

Negative yields have no economic justification at a credit risk level, since there is no negative probability of default. Nor do they have it at the level of the Eurozone: they are simply the symmetrical effect of an overshooting on other countries.

Certainly, countries enjoying a negative yield on their bonds could be tempted to resist the introduction of a European safe asset. However, it must be stressed that, while representing a short-term advantage, in the long run negative yields weaken the balance sheet structure of systemic national financial players, especially pension funds and insurance companies.

Since the 2010 crisis, several proposals have been put forward by both economists and politicians. Initially, these proposals mainly focused on the creation of ‘Eurobonds’ collectively guaranteed by member countries, thus involving a commitment to mutualisation. Thereafter, given the lack of a political consensus on mutualisation among Eurozone countries, the focus shifted to reform projects entailing the least amount of public guarantees possible. For a wide-ranging overview of the main single proposals, we suggest Claessens et al. (2012), Leandro and Zettelmeyer (2018a), and Giudice et al. (2019).

All the proposals made so far can be seen as particular combinations of pooling, tranching and public guarantees, with the shared purpose of achieving risk filtering.

A first type entails a mix of pooling and tranching without any form of public guarantee (Brunnermeier et al., 2011, 2017; ESRB, 2018). In this case, pooling precedes tranching, and the safe asset can be issued by a financial intermediary (public or private) which issues securities in two tranches: a senior one (European Senior Bonds, ESB) and another one subordinate to it (European Junior Bonds, EJB).

It is evident that what determines the degree of safety of the ESB is the proportion between ESB and EJB tranches. However, on the other hand it also entails the potential insecurity of the EJB. Hence, given the nature of ESBs, in the event of a systemic crisis, with bankruptcies of various countries, it is less certain that they would remain safe (De Grauwe & Ji, 2018; Gabor & Vestergaard, 2018).

A second type of scheme reverses the temporal order between tranching and pooling. Monti (2010), Juncker and Tremonti (2010) and Wendorff and Mahle (2015) put forward the first national tranching proposals, thus creating a senior debt segment and another one subordinate to it. Recently, Leandro and Zettelmeyer (2018a) and Giudice et al. (2019) have developed the national tranching solution.

The main weakness shared by the above proposals is the possibility that, in the case of systemic or idiosyncratic shocks, even a well-disciplined country may be unable to finance the rollover of junior bonds. Moreover, self-fulfilling expectations of default could arise because of the way in which public debt is issued.

In short, these proposals may be able to create safe assets (strengthening the senior tranche of each participating country more efficiently than in the previous case), but for the same reason they may weaken the liquidity of junior bonds and hence the solvency of the Member States that issue them.

There is a third, and different, type of Eurobond, which eliminates tranching and brings into play a common issuer (Leandro & Zettelmeyer, 2018b). In this case, the safe asset is issued by a public or semi-public common issuer with adequate capital endowment. Leandro and Zettelmeyer (2018b) analyse two variants of this third type.

There is finally a fourth type: bonds issued by a Eurozone budget (Ubide, 2015; Zettelmeyer, 2017). The strength of bonds of this type would be the Eurozone’s guarantee of repaying the debt issued in addition to having created an initial form of common European fiscal policy. The main weakness—which we are currently experiencing—is that current political conditions allow the issuance of an initially very limited amount of assets of this kind, given the total mutualization of risk that they imply, as well as the initial limitedness of the common tax revenues.

As evidenced by this brief survey, the ‘Eurobond puzzle’ seems to resist straightforward synthesis. In particular, the main goal of all tranching proposals, namely risk filtering, tends to transform itself surreptitiously into the goal of a translation of risk, to be overpaid by the more indebted nations.

One can then argue in favour of less ‘market-centred’ forms of safe asset issuing. But this option incurs the problem of the nature and the amount of the public guarantees required. The adequacy of a capital endowment depends also on the risk actually transferred to the agency charged with issuing the safe asset, i.e. it depends on the efficiency with which that agency performs the risk-filtering function.

On the one hand, there are the ‘market-centred’ proposals with a low degree of risk socialization, but which are possibly ‘unsafe’. On the other hand, there are ‘public-guarantees’ solutions, which are clearly safer but involve some form of risk sharing.

The policy problem to solve is therefore as follows: what are the conditions under which one can conceive a ‘safe agency’ issuing an adequate quantity of common safe assets (i) without requiring any form of preferred clause; (ii) without requiring any form of tranching; (iii) without requiring any form of mutualization; (iv) with a capital endowment minimizing public guarantees?

Until now, the option that has come closest to solving the puzzle is the one presented by Dosi et al. (2018). According to their proposal, the ESM should guarantee the sovereign debts of the Member States through the combined provision of a recapitalization and the establishment of an insurance scheme that guarantees the new ESM issues. This insurance scheme would have the advantage of defining a premium with respect to the risk of each country, thus avoiding mutualisation. In this way, the new ESM would support the full transition from national debt to a single Eurozone public debt with a single yield curve for all countries.

In this regard, we can imagine an even more structural institutional response, i.e. a European Debt Agency that, by leveraging on an insurance scheme, can perform systematic risk-filtering by lending to national borrowers according to perpetuity schemes.

This is the topic of the next section.

## The European Debt Agency

### Institutional framework

An adequate institutional vehicle at Eurozone level (to be established or which already exists) is chosen. Eurozone Member States are its main shareholders. The shared ownership could be established in accordance with paragraph 2 of article 122 of TFUE. It could initially rely on an ‘enhanced cooperation vehicle’ (art. 20 TUE and 326 et seq. TFUE). This vehicle could employ the resources already assigned to the ESM (Perillo, 2020). Let us call it the ‘European Debt Agency’ (henceforth ‘Debt Agency’).

Based on an adequate insurance scheme equivalent to a solvency capital endowment, the Debt Agency (i) collects liquid funds on markets by issuing plain vanilla bonds with finite maturity; (ii) uses these funds to finance Member States with infinite maturity loans.

The Debt Agency does not purchase outstanding debt on secondary markets. It finances Member States’ new debts with loans, either to cover new public deficits or to refinance expiring bonds. These new loans are meant to be repaid according to an irredeemable mortgage scheme, as if they were perpetuities. Put otherwise, the Debt Agency enables Member States to finance themselves without directly issuing perpetual bonds (difficult to sell on the markets), but to enjoy the fundamental advantage of the latter, which is a substantial alleviation of the terms of repayment.

The Debt Agency’s loan is not a negotiable security, but a loan held indefinitely on the records of the Debt Agency. This implies that it is analogous to a perpetuity structurally hedged against liquidity risk.^3^ Indeed, the main purpose and the truly distinctive feature of this operating framework consists in immunizing Member States’ fiscal budgets from market liquidity risk, aligning the debt cost to ‘fundamental risk’ (i.e. credit risk) alone.

For this reason, and also because of the presence of adequate solvency capital endowment based on an insurance scheme (see Sect. 4 below), no seniority clause is necessary to support the creditworthiness of the Debt Agency. This, at least formally, avoids a dualistic situation between debt in the Debt Agency and floating debt on the markets, i.e. it avoids a surreptitious resurgence of the cleavage or ‘juniority’ effect caused by tranching (see Sect. 2).

Since by design the Debt Agency filters the market liquidity spread risk, it can receive from each Member State a periodic instalment which is recalculated each year considering only Member State fundamental risk.^4^ This means that the Debt Agency does not involve any form of mutualisation. The overall flow of instalments, net of legal provisions, thus enables the Debt Agency to remunerate its bondholders at a rate in line with its high rating, and most of all with an ECB long-term rate.

Indeed, the ECB will stabilize the overall Debt Agency mechanism primarily by remunerating the Debt Agency’s reserves at a long-term directory rate, and secondarily by declaring its willingness to directly buy the Debt Agency’s bonds on the primary market. These two provisions would have the effect of aligning the Debt Agency’s bond yield to that rate, which will be lower than the average of the fundamental cost of each Member State (for the cost of the underlying portfolio see Appendix A.4).

This would be a new and perhaps more appropriate goal for the ECB, which could replace the ongoing purchase programmes (PSPP and PEPP) and more efficiently achieve the same goal of regulating the yield of national (and EU) debts, while at the same time avoiding all the formal criticism that has been made of those programmes.^5^

The Debt Agency would thus reach its financial equilibrium at conditions more advantageous than those of any portfolio manager on the market.

Even if the Debt Agency does not require the granting of ‘preferred creditor’ status, since the safety of the vehicle is obtained by means of its intrinsic architecture, this framework should nonetheless be completed by adequate provisions, such as:

1. independent governance of the Agency, in order to prevent or manage potential conflicts of interest (moral hazard);
2. compliance with the supervision standards established for the SSM (Single Supervisory Mechanism) and the European prudential regulation;
3. ex-ante budget control rules for Member States, and the design of recovery plans for the management of the debt conferred to the Agency in the case of forbearance, according to a regulatory framework to be established by EU Institutions.

To sum up: by acting as a ‘protective gap’ between the Member States and the markets, the Debt Agency allows

1. Member States to pay an instalment linked to their fundamental risk and only to that;
2. markets to rely on an asset from which liquidity risk and global market sentiment have been filtered, and which is consequently more secure than any possible portfolio;
3. systemic financial actors to overcome a safe asset shortage which makes it difficult for them to manage their business cycle (for banks, the liquidity cycle; for insurers and pension funds, the management of guarantees on returns to investment);
4. the Eurozone to behave as a homogeneous actor issuing a common bond which nonetheless does not need any kind of mutualization;
5. progressive dissolution of the ‘doom loop’ that at present links the solvency of Member States to that of the respective banking systems, and vice versa.

The Debt Agency bond does not imply mutualization, but it enjoys a ‘pooling effect’ (see below) that structurally reduces its risk. The Debt Agency thus acts as a ‘synthetic treasury’ issuing a common bond that can be bought by the ECB without infringing its commitment not to ‘favour’ any Member State, which is the raison d’être of the capital key rule.

The fact that the operation of the Debt Agency does not imply any kind of mutualization does not mean that it is incompatible with it, or even with a mixed regime of non-mutualized and mutualized loans (by means of separated sub-portfolios), as detailed below (Sect. 3.4).

### ALM considerations

**Fig. 1:**
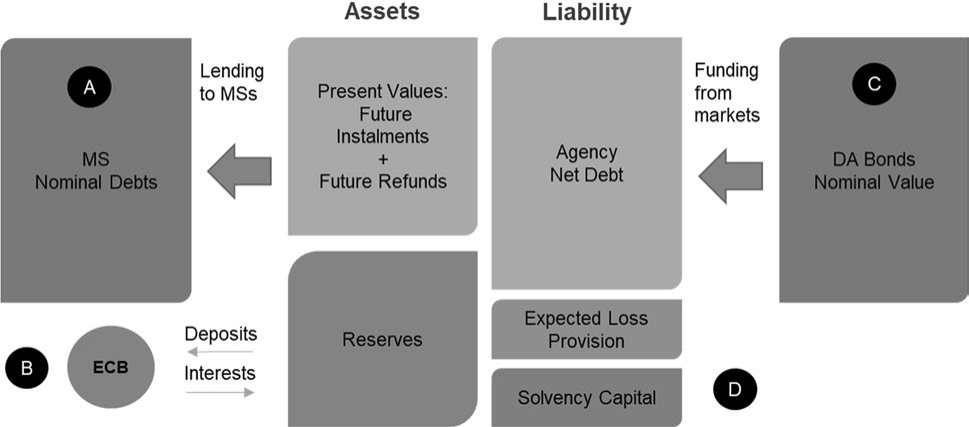
Debt Agency’s balance sheet structure

The following scheme itemizes the overall structure of the balance sheet of the Debt Agency:

(A) Lending facilities to Member States, the cost of which is based on an irredeemable mortgage scheme.
(B) Accumulated reserves, which take the form of remunerated deposits within the ECB.
(C) Debt Agency issuances, which take the form of indexed bonds with finite maturity.
(D) Solvency capital, which takes the form of an insurance scheme (Fig. 1).

#### The balance sheet asset side

Subject to EU budget control rules, and upon decision of the Member State, the Debt Agency finances with a loan the Member State’s current budget deficit or the repayment of expiring bonds.

Loans to Member States are granted against funding costs in line with the Agency’s fundamental risk. Loans have the implicit option of an infinite roll-over clause which guarantees to renew each Member State’s debts indefinitely. This implicit option is priced as a cost of an irredeemable mortgage scheme considering expected default probabilities.

The Debt Agency computes expected cumulative default probabilities (CDPs). As we will see in more detail in Sect. 3.4, the Debt Agency can compute instalments of the irredeemable mortgage scheme offered to each Member State and build up excess reserves that take the form of a remunerated deposit at the ECB.

Because the Debt Agency’s pricing for each Member State is invariant, the overall effect of the ECB remuneration is such that the present value of future instalments plus accumulated reserves always equals the outstanding debt of the Debt Agency:

$$\begin{aligned} \text{Outstanding Debt} = \left\{ \begin{array}[c]{c} \text{Present Value of Future Instalments}\\ +\\ \text{Accumulated Reserves} \end{array} \right\} ,\quad \text{for t } \ge 0. \end{aligned}$$

#### The balance sheet liability side

The Debt Agency issues indexed bonds of finite maturity on the primary market. The bond portfolio maturity mix will depend on investors’ preferences in the market.

The indexing scheme follows the reference long-term interest rate which the ECB applies on the deposit reserves of the Debt Agency. During auctions, the ECB will ensure alignment of the bid-ask price with the reference rate. Therefore, investors with different maturity preferences will enjoy the ‘legal certainty’ indexing mechanism for as long as they need in matching their intertemporal liability structure.

Since all maturity bonds are indexed at the same interest rate used to price them, the auction price will be aligned as closely as possible with their nominal principal. Consequently, the liability fair value corresponding to the bonds outstanding equals their nominal value (the Debt Agency will then value its liability at amortized cost).

To sum up:

1. Asset side: the fair value of the credit portfolio is computed with the reference rate recognised by the ECB, and the Debt Agency reserves deposit is remunerated by the ECB with the same reference interest rate;
2. Liability side: the Debt Agency’s liabilities are valued at amortized cost by considering an effective interest rate in line with the indexing mechanism.

As a consequence, the asset side and the liability side always move in the same direction, and the Debt Agency enjoys a situation of ‘near natural hedging’ (see Appendix A.3, prop. 12).

### Solvency capital

In its operating model, the Debt Agency conceives the default event of a Member State in terms of a ‘forbearance process’ whereby the Member State undergoes a period of restructuring. During this period, the revised level of the fundamental instalment becomes too high to be affordable by the single Member State; hence its payment must be suspended.

In order to preserve the Debt Agency’s financial equilibrium, an insurance scheme is instituted. Member States that are not in forbearance are required to pay an extra premium (additional bps), which the Debt Agency will reserve and treat as an allowance to cover the temporary deficiency of instalments necessary to ensure its financial equilibrium.

The insurance scheme is managed as a self-insurance plan. The insurance premiums are computed by considering ‘stressed default probabilities’ (calibrated in line with a stressed scenario exercise available from the European Systemic Risk Board).

In the case of ‘default’ by a Member State, the insurance scheme will provide an amount of capital equivalent to the present value of instalments due from a Member State in state of forbearance for an estimated suitable period (see Appendix A.5, eq. 17) We have shown that the Debt Agency framework can perform terms-transformation without incurring ALM risks, doing so through an appropriate mechanism of risk transfer pricing supported by an active role of the ECB.^6^

In the next section we describe the analytical foundations of the operating model of the Debt Agency.

### Pricing of the irredeemable mortgage scheme

Each Member State *i* is assigned to a specific *credit risk class* *j* based on its creditworthiness according to agreed methodologies and risk models (e.g. based on the above-mentioned revision of the stability and growth pact). It is then possible, under given analytical conditions (see Appendices A.1 and A.2), to compute the expected present value of a unitary perpetual annuity ${\tilde{a}}_{j}$. The corresponding annual instalment cost of the irredeemable mortgage granted to a Member State with nominal debt equal $d_{i}$ is then given by:

1 $$\begin{aligned} c_{f,ij}=\frac{d_{i}}{{\tilde{a}}_{j}} \end{aligned}$$

with $d_{ij}$ representing the conferred debt of the Member State to the Debt Agency.

As mentioned, the calculation at time *t* of the value of ${\tilde{a}}_{j}$ can be done by means of different methodologies such as e.g. by filtering fundamental sovereign debt credit spreads from market yields timeseries (Paniagua et al., 2017) or alternatively by resorting to a by-design transition matrix built on purpose in order to preserve some fundamental desired theoretical properties (see Appendix A.1).

The specific feature of the formula (1), that we label “idiomatic fundamental price”, is that each Member State pays for the risk inherent to the specific credit risk class *j* to which it is assigned, without involving any form of solidarity or mutuality among Member States of different credit risk classes. From the above formula we obtain ${\tilde{\pi }}_{j}=1/{\tilde{a}}_{j}$, that can be seen as a risk-adjusted discount rate attributed to the credit risk class of the Member State. Thanks to the irredeemable nature of the loan granted by the Debt Agency, the instalment corresponds to the risk-adjusted interest that a Member State of credit risk class *j* has to pay annually to finance its debt based on its creditworthiness.

If the Debt Agency prices at time *t* its loans portfolio using an irredeemable annuity ${\tilde{a}}_{W}(t)$ measure computed as the weighted average of the annuities of the credit risk classes considered using the relative capital weight exposure for each class, then the agency intertemporal equilibrium is assured in expectation (see Appendix A.3). In fact, considering *n* credit risk classes, the following Eq. (2) holds true if the Debt Agency can determine a fair overall instalment $I_{W}(t)$ for its portfolio such that:

2 $$\begin{aligned} TD=I_{W}(t){\tilde{a}}_{W}(t)=I_{W}(t)\sum _{j=1,\ldots ,n}w_{j}{\tilde{a}}_{j} \end{aligned}$$

where $TD=\sum _{j=1,\ldots ,n}d_{j}$ represents the total amount of loans granted to the Member States, with $d_{j}=\sum _{i=1,\ldots ,k}d_{ij}$ the overall amount of debt for the credit risk class *j* and $w_{j}=d_{j}/TD$. Notice that according to Eq. (2) the instalment attributed to each debt class *j* is proportional to the corresponding nominal debt:

$$\begin{aligned} c_{j}=I_{W}(t)w_{j}=I_{W}(t)\frac{d_{j}}{TD} \end{aligned}$$

and on the same line, the instalments $c_{ij}$ required to the Member State *i* in class *j* : 

$$\begin{aligned} c_{ij}=I_{W}(t)w_{i}. \end{aligned}$$

It is easy to verify (see Appendix A.3), that the total payment $I_{W}(t)$ obtained under the above equilibrium condition is structurally lower than the amount due if the total instalment collected by the agency was equal to the algebraic sum of the idiomatic fundamental price of each Member State, $I_{F}(t)=\sum _{ij}c_{f,ij}$. This is attributable to a "pooling effect" involved with the mathematical properties of the problem. It turns out that, if we let *l* and *h* label respectively the low and the high risk classes, we have:

$$\begin{aligned} c_{f,l}<c_{l}<c_{h}<c_{h,l} \end{aligned}$$

that is, under the solution of Eq. (2), instalments are less differentiated, with low risk debt classes paying partly more and higher risk debt classes paying partly less than they would under the idiomatic solution. This result has mutualistic flavor and produces an overall risk mitigation benefit. Besides, this result also implies, for each year of observation, potential improvements in the rating of the worst classes.

However, if the Debt Agency charged each Member State using the formula (1), it would collect additional capital available for solvency purposes and would create value for its stakeholders. Moreover, it would minimize the potential moral hazard, of enhancing debt expansion of the worst risk class countries.

The question of the overall cost distribution among Member States is then crucial in order to identify the desirable benefits that can be drawn by an optimal setup of the Debt Agency’s. We want to emphasise that the overall cost of the portfolio obviously depends on the cost distribution rule adopted to charge each credit risk class.

It is however possible to “bridge” the two solutions. To avoid moral hazard and charge each Member State according its idiomatic risk, the excess cost of $I_{F}(t)-I_{W}(t)\ge 0$ could possibly be allocated among credit risk classes without compromising the agency’s financial equilibrium, giving rise to the following proposition. In Appendix A.6 we show that it is possible to rebalance the individual instalments for each rating class to new values $c_{j}^{\prime }$, minimizing a distance between their present value (adjusted for risk) and the nominal debt ($d_{j}$). More precisely the following two Propositions hold.

#### **Proposition 1**

*Assuming a risk perspective and designating as* $c_{f,j}=\sum _{ij}c_{f,ij}$ *the total idiomatic cost for the credit risk class* *j*, *the agency optimal cost allocation should be so that the present value of the risk adjusted class cost* $w_{j}^{\prime }I_{W}(t){\tilde{a}}_{j}$ *be as close as possible to* $d_{j}$. *Thus*, *we want to find new weights* $w_{1}^{\prime },\ldots ,w_{j}^{\prime } ,\ldots w_{n}^{\prime }$ *that satisfy the following minimization problem*:

$$\begin{aligned} \min _{w_{1}^{\prime },\ldots ,w_{j}^{\prime },\ldots w_{n}^{\prime }}\sum _{j=1,\ldots ,n} (w_{j}^{\prime }I_{W}(t){\tilde{a}}_{j}-d_{j})^{2} \end{aligned}$$

*subject to* $$\sum _{j=1,\ldots ,n}w_{j}^{\prime }=1.$$

#### **Proposition 2**

*The solution of the problem gives* $w_{j}^{\prime }I_{W}(t)=c_{f,j} -(I_{F}(t)-I_{W}(t))\frac{{\tilde{\pi }}_{j}^{2}}{\sum _{j=1,\ldots ,n}{\tilde{\pi }} _{j}^{2}}$, *i.e. the excess cost can be redistributed to each rating grade class by subtracting a fixed amount of* $(I_{F}(t)-I_{W}(t))/n$ *from the corresponding idiomatic cost* $c_{fj}$.

The proof of both propositions is supplied in Appendix A.6.

The reduction of the instalment illustrated above is equivalent to a premium that is inversely proportional to the riskiness of each Member State, throughout the servicing cost of debt ${\tilde{\pi }}_{j}$. Enjoying a convexity effect, the surplus obtained thanks to the pooling effect is distributed in order to compensate the Member States with higher risk. At first glance, this may appear as an exception to the principle of strict proportionality governing the Debt Agency’s relationship with the Member States. But it would be an exception stemming by design from the technical modus operandi of the Debt Agency, and not from political decisions.

The funding cost structure weighing on a single Member State has been computed by considering its specific “fundamental risk”, which is obviously the result of many factors, such as the debt-to-GDP ratio, the current deficit and all the other macroeconomic variables that influence Member States’ creditworthiness. By leveraging the potentially irredeemable nature of sovereign debts, we have intended to price the overall cost by means of an amortizing scheme according to which every single Member State pays for an infinite period of time only a risk-adjusted interest, if necessary recalculated on a periodic basis. There is in fact no conclusive economic reason why a Member State should redeem its debt with the Debt Agency at a fixed date, whereas the Debt Agency can manage its own debt renewals and deadlines autonomously by resorting to new debt collected in the market and financed at lower costs, thanks to its higher credit standing.

Nevertheless, we would like to point out that the basic solution developed in this framework is fully non-mutual. In this sense, Debt Agency can operate without requiring any modification of the treaties. However, as mentioned above, there is nothing to prevent Debt Agency from proceeding according to mixed methods. Implementing formal political decisions, the Debt Agency could build segregated (mutualized and non-mutualized) sub-portfolios. The distinction in the treatment of national debts would rest then on political grounds. By way of example, the Debt Agency could apply a mutualisation scheme to national expenses carried out in the framework of European infrastructure cooperation programs, while non-mutualisation would continue to be applied to debt expansions linked to strictly national fiscal policies.

$$\begin{aligned} ***\end{aligned}$$

In next section we propose a quantitative exercise sustaining our framework by considering a credit portfolio ${\mathbf {d}}^{T}=[d_{1},\ldots ,d_{j},\ldots d_{n}]$ of total holding $TD=\sum _{j=1,\ldots ,n}d_{j}$, held by the Debt Agency and diversified in *n* different credit risk classes of obligors, each credit risk class *j* with total exposure $d_{j}$. Note that the last *n*-th class stands for a default absorbing status and has at the inception $d_{n}=0$. In projecting portfolio expected values, we assume a portfolio with *infinite granularity of obligors* in each credit risk class, so that the exposure to each single obligor of class *j* is infinitesimal and we justify this methodological assumption in light of the assured configuration of the mortgage scheme adopted. Neglecting Debt Agency operating expenses, we will highlight five distinctive aspects of our framework^7^:

1. the *credit risk migration model* as an exemplification upon which we can derive our propositions and perform a numerical exercise (Appendix A.1);
2. the derivation of the *perpetual annuities and the fundamental risk measure for each credit risk class*, following an irredeemable amortization scheme (Appendix A.2);
3. the *pricing of the Debt Agency portfolio* (Appendix A.3);
4. the financial *Debt Agency intertemporal equilibrium* of the under expected conditions (Appendix A.4);
5. the *required risk-capital for solvency purposes* in the form of an insurance scheme (Appendix A.5).

## Numerical application

The numerical application of the model is based on a simplified *TTC* *transition matrix*. We estimated this matrix by using publicly available data^8^ of rating grades assigned to sovereign debts by Credit Rating Agencies in the period 1993–2015.^9^ This period has been chosen to include aspects of major institutional changes (e.g. events such as the introduction of the euro or the Eurozone sovereign debt crisis).

### Perpetual annuities and insurance scheme

On the basis of the assumptions and formulas presented here, a projection has been performed to show the long-term evolution (1000 years) of the economic balance sheet of the Debt Agency under different LGD hypothesis and exposure $TD=100$.

The *TTC* transition matrix has been decomposed as illustrated in Eq. (5). A fundamental characteristic of the matrix is that it exhibits an almost zero 1-year default probability for ratings from AAA to BBB. This is not surprising, since investment-grade ratings in the medium-term should only be subject to migration risk (Table 3).

**Table 3:** Estimated TTC transition matrix

	AAA	AA	A	BBB	BB	B	CCC	D
AAA	0.9599	0.0401	0	0	0	0	0	0
AA	0.0179	0.9107	0.0643	0.0071	0	0	0	0
A	0	0.0281	0.8989	0.0730	0	0	0	0
BBB	0	0	0.0528	0.8746	0.0561	0.0132	0.0033	0
BB	0	0	0	0.0490	0.8529	0.0784	0.0131	0.0065
B	0	0	0	0	0.0706	0.8853	0.0294	0.0147
CCC	0	0	0	0	0	0.3846	0.4231	0.1923
D	0	0	0	0	0	0	0	1

We simulated a portfolio of sovereign central government debts the mix of which, ordered by rating grades, corresponds to the Eurozone Member States in 2018 (ECB, 2019):

$$\begin{aligned}{}\begin{array}[c]{lllllll} \text{AAA} &{} \text{AA} &{} \text{A} &{} \text{BBB} &{} \text{BB} &{} \text{B} &{} \text{CCC}\\ 25\% &{} 34\% &{} 12\% &{} 26\% &{} 3\% &{} 0\% &{} 0\% \end{array}. \end{aligned}$$

Assuming a rate $\pi =50$ bps, the table below displays the values of perpetual annuities and corresponding annual costs for 100 units of debt by rating grades class under different hypotheses of loss given default (LGD). Note that for $LGD=0$ the perpetual annuity corresponds to the value of $1/\pi =1/0.005=200$ for unit of debt (Tables 4 and 5).

**Table 4:** Unitary perpetual annuity by LGD value

LGD	0%	50%	60%	70%	80%	90%	100%
AAA	200	140	132	125	119	113	108
AA	200	130	121	114	107	102	96
A	200	121	113	105	98	92	87
BBB	200	110	101	93	87	81	76
BB	200	95	86	78	72	67	62
B	200	85	76	69	63	58	54
CCC	200	62	55	49	44	40	37

**Table 5:** Annual cost per 100 of capital by LGD value

LGD	0%	50%	60%	70%	80%	90%	100%
AAA	0.50	0.71	0.75	0.79	0.84	0.88	0.92
AA	0.50	0.77	0.82	0.87	0.93	0.98	1.03
A	0.50	0.82	0.88	0.95	1.01	1.08	1.14
BBB	0.50	0.90	0.98	1.07	1.15	1.23	1.31
BB	0.50	1.05	1.16	1.27	1.38	1.50	1.61
B	0.50	1.18	1.31	1.44	1.58	1.71	1.85
CCC	0.50	1.60	1.82	2.04	2.26	2.48	2.70

We have also addressed the question of how costly should be the insurance scheme to manage the risk of restructuring Member States (forbearance).

To enable quantification of such a cost, we relied on the stressed scenario as elaborated by the ESRB (2018), which gives an estimation of the overall 5-years stressed default probability of a European portfolio of sovereign debts, weighted by their outstanding amounts. We used this value for the Debt Agency’s credit portfolio to calibrate stressed transition matrix in order to obtain a coherent PDs term structure to compute the insurance premiums according to the formula (17). From stressed cumulative default probabilities we could then infer the implied forward default probabilities needed. Tables 6 and 7 report respectively stressed cumulative default probabilities and an estimation of the premiums by rating grade class.

**Table 6:** CDP stressed

Years	1 (%)	5 (%)	10 (%)	15 (%)	20 (%)	25 (%)	30 (%)
AAA	0.15	20.0	57.7	79.3	90.2	95.4	97.8
AA	1.0	31.6	66.4	83.7	92.3	96.4	98.3
A	2.8	40.6	72.0	86.5	93.7	97.0	98.6
BBB	8.4	50.9	77.5	89.2	94.9	97.6	98.9
BB	19.1	62.6	83.3	92.1	96.3	98.3	99.2
B	27.3	68.8	86.3	93.5	97.0	98.6	99.3
CCC	50.1	78.8	90.7	95.6	97.9	99.0	99.5
D	100	100	100	100	100	100	100

**Table 7:** Premium per 100 of capital by LGD value

LGD	0%	50%	60%	70%	80%	90%	100%
AAA	0.042	0.060	0.063	0.067	0.071	0.074	0.078
AA	0.047	0.073	0.078	0.083	0.088	0.093	0.099
A	0.053	0.087	0.094	0.101	0.107	0.114	0.121
BBB	0.061	0.111	0.121	0.131	0.141	0.150	0.160
BB	0.076	0.160	0.177	0.193	0.210	0.227	0.243
B	0.087	0.206	0.230	0.253	0.277	0.301	0.324
CCC	0.128	0.412	0.468	0.525	0.581	0.637	0.693

### A counterfactual analysis

In previous paragraphs, we showed how to calculate the MSs’ instalments for different LGDs starting from a 50-bps risk-free interest rate, as well as the operating foundations of the Debt Agency’s insurance scheme.

After these prospective exercises, which should give an idea of how the Debt Agency works conceptually, it may be useful to perform a retrospective exercise intended to show how the Debt Agency could have worked historically. The aim of the counterfactual exercise is to evaluate the effects of the European Debt Agency in terms not only of a potential mitigation of the cost of debt, but above all of the systematic alignment of that cost to the fundamental credit risk, for all member states.

In other words: what could have been the dynamics of the sovereign debt service in the eurozone if, since its inception, that goal had been pursued in a coordinated, non-competitive way? Accordingly, we performed a simulation starting from the introduction of the single currency up to the end of 2015, because from 2016 on the prices of government bonds have been strongly influenced by the operations of the ECB (PSPP and PEPP).

For this purpose, the following simplifications were adopted:

- The historical series of 10-year government bond yields was used as a proxy for the cost of debt service because it is generally deemed to be the one most correlated to the average issuance cost of sovereign debts.^10^
- The main countries of the Eurozone in terms of exposure (Germany, France, Italy and Spain) were considered separately; the rest were aggregated into a residual group (“Euro-Others”).
- In order to avoid distortions in measuring the discount rate used in pricing, Greece was excluded, being considered in default since 2012.
- The discount rate $\pi$ for the computation of the idiomatic cost of each Member State was calculated according to an iterative procedure^11^ that started from calculation of the average portfolio yield. For each time-step of the historical series, the yield was obtained by weighting each country’s yield with the corresponding debt recorded on that date.^12^ The procedure allowed identification of a ‘risk sensitive’ discount rate with respect to the ‘global risk factors’ (liquidity risk plus global market sentiment) inherent to the Eurozone as a whole, implicitly captured in the historical series of the yields. This procedure should have given a reasonable proxy for the discount rate used by the Debt Agency since 2002.
- The idiomatic cost associated with each Member State was calculated on the basis of the Fitch rating corresponding to each record of the historical series.^13^

**Fig. 2:**
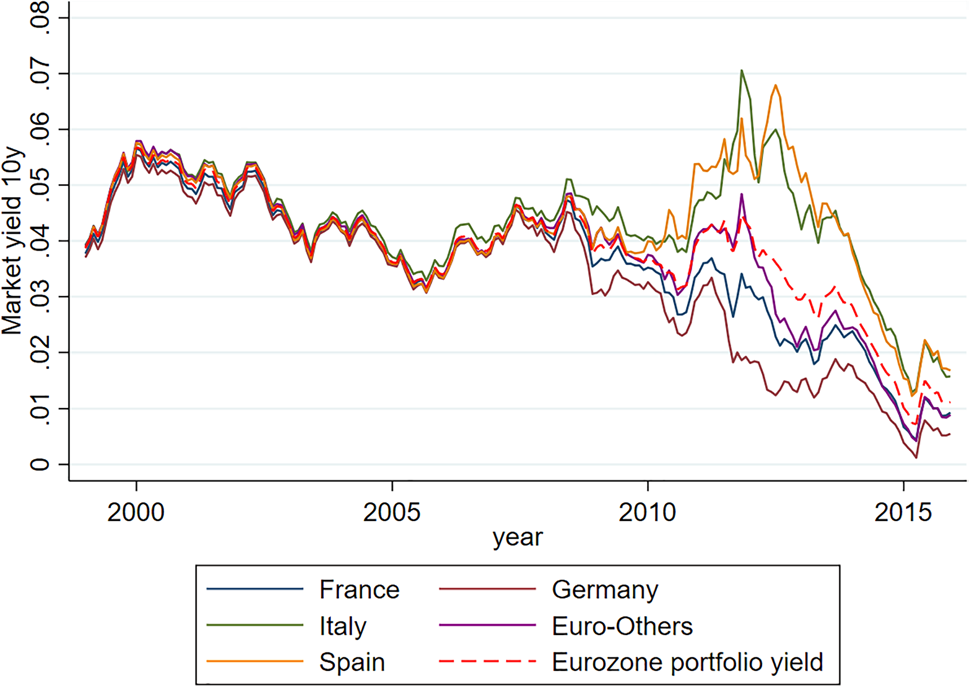
Historical series of yields (DE, FR, IT, SP, Euro-Others, synthetic yield)

Figure 2 illustrates the historical trends of the yields related to the aggregates of the series.

Figure 2 clearly shows that, after the inception of the euro in 2011, all Member States enjoyed an advantage largely determined by expectations of a prospective mitigation of the servicing cost of the debt. By contrast, since the sovereign debt crisis of 2011–2012, a process of divergence has occurred which has had an adverse impact on some countries, due to negative market expectations as to the sustainability of the single currency. As the graph shows, compared to the average portfolio yield (dotted line) a pattern emerges which is characterized by a ‘divergent symmetry’ (‘symmetrical divergence’) between countries with high credit rating (primarily Germany) and countries with a tight budget constraint (especially Italy). This resulted, for some Member States, in a cost of debt service higher than their actual fundamental risk, and conversely in a corresponding lower cost for others, despite the emergence of a significant contagion risk with potential disruptive effects on the overall fiscal structure of the eurozone.

What would have happened if the cost of servicing the debt of these Member States had been calculated on the basis of the idiomatic pricing scheme proposed here?

**Fig. 3:**
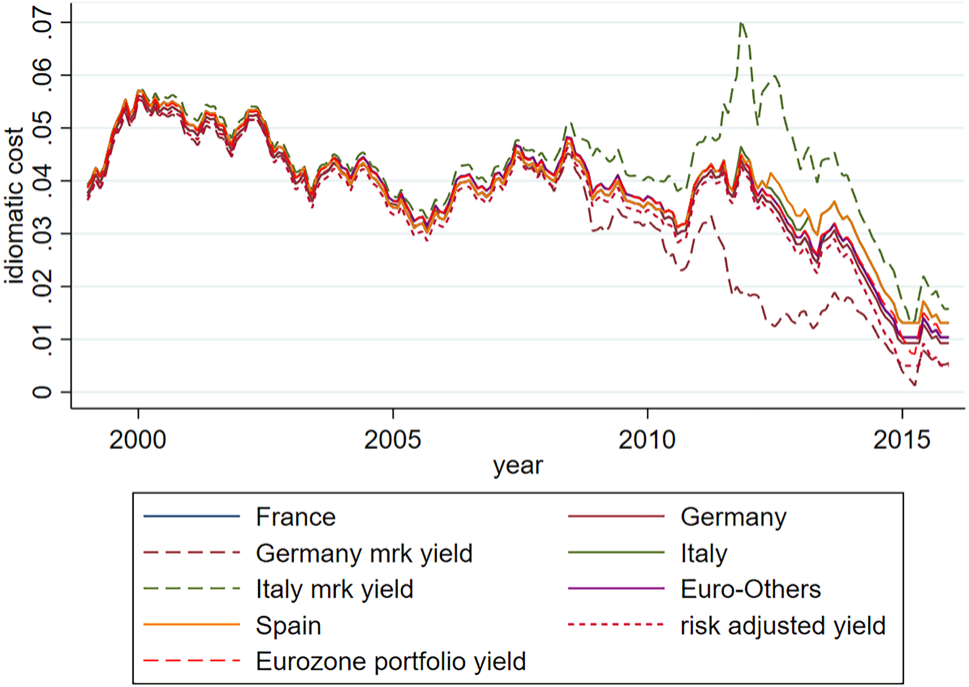
Idiomatic cost DE, FR, IT, SP, Euro-Others; yield IT, yield DE

Figure 3 shows the historical series of idiomatic costs recalculated on the hypothesis of a Debt Agency operational since 2002, and compares them with the cost associated with Germany (lower yellow line) and Italy (upper green line). As can be seen, these costs are ‘risk sensitive’: they are certainly affected by non-systematic changes in the macroeconomic market conditions, but they are ‘hedged’ against the expectations endogenously formed on financial markets. Furthermore, these costs do not manifest ‘diverging symmetries’ in favour or against a particular Member State, since they are calculated on the assumption that a ‘systemic risk factor’ operates at the level of the entire eurozone.

**Fig. 4:**
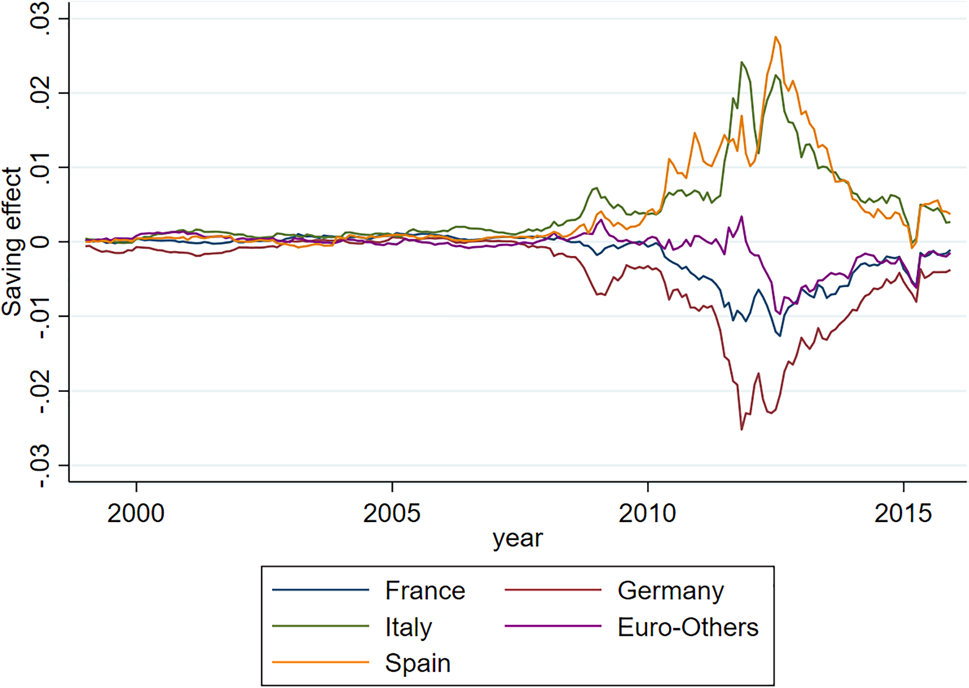
Saving effect DE, FR, IT, SP, Euro-Others

So, what would have been the ‘saving effect’ if a Debt Agency had already been in place when the single currency was adopted? Figure 4 shows the historical series of a variable equal to the market yield, net of the corresponding idiomatic cost (recalculated for each time-step in the series).

As can be easily observed, some MSs (notably Germany, France and Euro-Others) enjoyed a ‘premium’ by leveraging on the structural weakness of other MSs (notably Italy and Spain). This evidences the paradox of purely ‘market based’ valuations, i.e. the ‘market mispricing’ of the component linked to systemic risk. Had it been correctly considered, this component could not have justified such divergent patterns in the pricing of the debt of countries with structurally integrated economies, as demonstrated by the management of the pandemic crisis by the EU and the ECB. With the new rules at the base of the PEPP, the ECB has largely mitigated the ‘symmetrical divergence effect’ revealed by our counterfactual exercise. Our hypothesis is that the Debt Agency, working in structural synergy with the ECB, could mitigate the divergence in an even more structural way.

## Conclusions

In this paper we have presented the design and functioning of a European Debt Agency conceived as a market operator owned by the Member States. The Debt Agency, with its public mission of aligning the cost of public debt servicing of each Eurozone Member State with respect to its own fundamental risk, thus resembles a European institution more than an international entity.

Since the Debt Agency lends to Member States according to an irredeemable mortgage scheme and by funding itself on the market through the issuance of finite maturity bonds, it is entitled to issue a true common European safe asset. This is the reason why the ECB is fully justified in directly buying the Debt Agency’s bonds in order to guarantee that their yield is not superior to their nominal indexed rate.

The main objectives of the Debt Agency can be summarized as follows:

1. to provide the European financial system with a common public asset able to maintain a high rating even during extreme systemic crises;
2. to use the least amount of public guarantees possible;
3. to break the doom-loop between public debt and national banking systems;
4. to align the cost of Eurozone Member States’ public debts with their respective fundamental risk;
5. to smoothe and eventually stop the divergence dynamic on the sovereign debt market due to liquidity risk (as to these latter points, see the prospective and retrospective simulations in Sect. 4).

We may add a further point, i.e. the ability of the Debt Agency to become an accelerator of the integration process by managing the progressive shift from national to common debt (EU budget backed) issuance, and by facilitating the progressive enlargement of the Eurozone to all EU members.

The presence on the sovereign debt market of an asset structurally safer and more stable than any individual Eurozone sovereign debt will gradually reduce to the minimum—until it eliminates—the divergence dynamic on outstanding debt due to liquidity risk. This effect ensues from the diminishing proportion of outstanding debt with respect to the debt held by the Debt Agency. This divergence-absorption effect, jointly with the ECB’s capacity to align the Debt Agency’s bond yield to its nominal rate, can help in managing the interval between the inception of the Debt Agency and its complete absorption of the Member States’ debt.

Concerning the implementation of the Debt Agency, to be noted is that at the first stages there may be a potential ‘juniority effect’ on the outstanding debt that leads to an explosion of the spreads. However, there are several mitigating factors that can be mentioned:

1. because the Debt Agency buys the expiring debt on its renewal at its face value, the best strategy for each bond holder is to hold bonds to maturity;
2. should the outstanding bonds be traded, they would ‘compete’ with the Debt Agency’s newly issued bonds, which should be used first in a LIFO perspective (moreover, the Debt Agency can issue its bonds with different maturities according to the needs of the market);
3. since the ‘juniority effect’ necessarily diminishes with the progressive absorption of the outstanding debt in the Debt Agency, the ECB can act residually, as it already does with PEPP, in order to prevent speculative rallies on the outstanding debt.

Since the safe asset issued by the Debt Agency would enable national banks gradually to replace national debt with safe European debt, the European banking system would benefit in several ways. The main ones concern the availability of excellent collateral for their daily activities, the settlement of a lower level of capital given the reduction in the risk of the assets held, as well as the possibility for banks and financial institutions to rely on a benchmark asset that would help them in pricing evaluations.

The solution that we propose in this paper is a medium-term one, since its implementation requires some time. However, its design is such that any emergency debt issue performed in the meantime can be subsequently absorbed into its normal functioning. In other words, it can give not only a stabilizing horizon to the emergency measures to be adopted during these unprecedentedly difficult times, but also a horizon for a ‘new European normal’ based on the strengthening and acceleration of the integration process towards, if not fully federal, at least more cooperative UE institutions.

In fact, the Debt Agency could prove crucial for the implementation of a rollover strategy for the EU common debt. The Recovery Fund envisages a 30-year duration, with repayment beginning in 2028. However, in the absence of rollover each maturity implies a refinancing risk, which the Debt Agency instead structurally filters.

The Debt Agency thus constitutes an important tool within an incremental strategy towards a fully federal treasury. In particular, it allows the financing of national debts alongside the common one, while favouring a smooth transition from the prevalence of the former to the prevalence of the latter. Even within national debts, the Debt Agency could allow differentiated financing strategies: non-mutualistic for fiscal expansions linked to national needs, and mutualistic for fiscal expansions linked to cooperative policy goals.

In view of a normalization of relations with the markets, the renewed role of the ECB envisaged within the framework of the Debt Agency would enable the ECB to gradually divest the purchase programmes put in place up to now.

The implementation of the Debt Agency with a view to a more complete union could also be an opportunity to rewrite the Eurozone’s operating rules. The suspension of the stability pact and its desirable rewriting would in this case be linked to a reworking of the criteria with which to define the fundamental risk. A careful rewriting would keep moral hazard at bay whilst at the same time redefining the terms for collaboration among states and increasing the margins for autonomous and responsible management of national fiscal policies.

To conclude, the Debt Agency could facilitate the enlargement of the Eurozone, and therefore the completion of European integration. An overall debt management which allowed wider margins of autonomy for national fiscal policies and at the same time was able to count on the proactive action by the ECB as a common central bank, would remove many of the reasons that have so far kept advanced and healthy EU economies away from the Eurozone.

## Appendix

### A.1 Credit risk migration model

To measure the credit-standing migration risk to which each credit risk class is exposed, we propose a methodology with which to calculate perpetual annuities based on a theoretical *through-the-cycle transition matrix *to show the feasibility of the Debt Agency framework proposed. Given a *point-in-time transition matrix* $TM_{t}$ at time *t*, its generic element $a_{ji}$ represents the annual probability that an obligor of the credit risk class *j* in year *t* will pass to a credit risk class *i* in the following year. The matrix has dimension $n\times n$ and the elements of row *j*, $a_{j1},\ldots ,a_{jn}$ must sum to unity, since every obligor with rating *j* will certainly be assigned to some credit risk class $z\in$ $\left\{ 1,\ldots ,j,\ldots n\right\}$ from year *t* to $t+1$; including the case of being reassigned to the same class *j*. As a convention, the rows and the columns of $TM_{t}$ are ordered according to safety class, from the safest (conventionally labeled AAA) to the default (label D: default state). Following the standard diagonalization method for square matrices, we assume that the $TM_{t}$ matrix can be decomposed in a *Q* matrix and a $L_{t}$ diagonal matrix so that:

3 $$\begin{aligned} TM_{t}=QL_{t}Q^{-1}. \end{aligned}$$

The $L_{t}$ matrix depends on *t* and shows correlations with the business cycle^14^ in its elements $l_{j}(t)$. In particular we assume that these values depend on shocks of the business cycle according to the following generalized logistic function:

4 $$\begin{aligned} l_{j}(y)=\frac{\theta _{j1}}{(1+\theta _{j2}\exp (\theta _{j3}y))} \end{aligned}$$

with $$y\ $$a *single factor stochastic process* and parameters $\theta _{j,1,2,3}$ calibrated so that the expected value of the $l_{j}$ is $E(l_{j})=\lambda _{j}$, with $\lambda _{j}$ being the element *j* of the eigenvalues diagonal matrix $\Lambda$ in the decomposition:

5 $$\begin{aligned} TTC=Q\Lambda Q^{-1}. \end{aligned}$$

Since the decomposition is unique unless linear transformations, then *Q* represents the eigenvectors matrix of the above linear functional.

#### **Proposition 3**

*Given the filtered probability space* $({\varvec{\Omega }},{\varvec{\Sigma }},{\varvec{\digamma }} _{t},{\mathbf {P}})$, *the matrix TTC*, *interpreted as a* through-the-cycle matrix,^15^*is the expectation of the stochastic process* $\left\{ TM_{t}\right\} _{t\ge 0}$ *adapted to the natural filtration* ${\varvec{\digamma }}_{t}$ *generated by* *y*.

#### *Proof*

Take the expectation of $TM_{t}$ and substitute $E(l_{j})$ with $\lambda _{j}$:

$$\begin{aligned} TTC&=E(TM_{t})\\ &=E(QL_{t}Q^{-1})\\&=QE(L_{t})Q^{-1}\\ &=Q\Lambda Q^{-1} \end{aligned}$$

$$\square $$

Following this model, the *expected cumulative default probability* in the interval $\left[ t,t+m\right]$ is the linear operator given by:

6 $$\begin{aligned} E\left[ {{\mathbf{cdp}}}(t+m)-{\mathbf{cdp}}(t)\right] =Q(\Lambda ^{t+m} -\Lambda ^{t})Q^{-1}{\mathbf {v}} \end{aligned}$$

where ${{\mathbf{cdp}}}(t)$ is an *n*-components stochastic process, the *j*-th element of which, $cdp_{{\mathbf {j}}}(t)$, represents the cumulative default probability that an obligor of rating grade class $j=1,\ldots ,n$ will have defaulted by time *t*, with ${{\mathbf{cdp}}}(0)=0$ and ${\mathbf {v}}$ a null vector apart its last element equal to 1.

#### **Proposition 4**

*The process* ${{\mathbf{cdp}}}(t)$ *can be seen as a stochastic vector depending from the process* *y*. *Since the matrix* $L_{t}$ *depends deterministically from* *y*, *this guarantees that* ${{\mathbf{cdp}}}(t)$ *is measurable given the filtration* ${\varvec{\digamma }}_{t}$ *generated by* *y*.^16^

### A.2 Perpetual annuities and fundamental pricing

Since our primary goal is to price the fundamental risk of each obligor credit risk class *j*, henceforth we will use the matrix *TTC* as the reference risk metric to calculate obligors’ default probabilities under expectations. We assume that the valuation date occurs at time $t=0$ unless otherwise specified, which simplifies our notation, but all the following mathematical expressions can be easily rephrased in order to include index $t_{0}$ to fix any time reference in the interval $\left[ 0,t\right]$.

Under the condition (6), the survival probability vector in the interval $\tau \in [0,t]$ can be written as:

7 $$\begin{aligned} {\mathbf{sp}}(0,t)=E\left[ {\varvec{1}}-{{\mathbf{cdp}}}(t)\right] ={\varvec{1}}-Q\Lambda ^{t}Q^{-1}{\mathbf {v}} \end{aligned}$$

where ${\varvec{1}}$ is the unit vector. The *n*-th element of vector ${\mathbf{sp}}\left( 0,t\right)$ corresponding to the default state is null.

The expected present value of a vector of unitary annuity maturing at time *t* can be written as:

$$\begin{aligned} {\mathbf {a}}(0,t)=\sum _{\tau =0,1,\ldots t}\frac{1}{(1+\pi )^{\tau }}{\mathbf{sp}} (0,\tau ) \end{aligned}$$

where $\pi$ represents a common appropriate financial discount rate.^17^ Note that the components of vector ${\mathbf {a}}(0,t)$ are ordered decreasingly, with the highest rating grades corresponding to higher annuity values since the present value of a unitary annuity is proportional to the survival probability of the corresponding credit risk class and a null value for the vector’s last component. Using the expression of ${\mathbf{sp}}(t)$ in Eq. (7), we can rewrite:

$$\begin{aligned} {\mathbf {a}}(0,t)=\sum _{\tau =0,1,\ldots t}\left[ \frac{1}{(1+\pi )^{\tau }}\left( {\varvec{1}}-Q\Lambda ^{\tau }Q^{-1}{\mathbf {v}}\right) \right] . \end{aligned}$$

Now, by letting $\alpha =1/(1+\pi )$ and $\beta _{j}=$ $\lambda _{j}/(1+\pi )$, with $\lambda _{j}$ the *j*-th eigenvalue in matrix $\Lambda$, the above expression can be written:

$$\begin{aligned} {\mathbf {a}}(0,t)=\alpha \frac{1-\alpha ^{t}}{1-\alpha }{\varvec{1}} -QBQ^{-1}{\mathbf {v}} \end{aligned}$$

where *B* is a diagonal matrix whose *j*-th element is $b_{j}=$ $\beta _{j}\frac{1-\beta _{j}^{t}}{1-\beta _{j}}$. Since the terms in $\Lambda$ are $\lambda _{j}\le 1$, it follows $\alpha ,\beta _{j}\in (0,1)$. By taking the limit for $t\rightarrow \infty$ we obtain the following perpetual annuity formula:

8 $$\begin{aligned} {\mathbf {a}}(0)=\lim _{t\rightarrow \infty }{\mathbf {a}}(0,t) =\frac{\alpha }{1-\alpha }{\mathbf {1}}-QB^{\prime }Q^{-1}{\mathbf {v}} \end{aligned}$$

with $B^{\prime }$ a diagonal matrix with *j*-th element $b_{j}^{\prime } =\frac{\beta _{j}}{1-\beta _{j}}$. The vector ${\mathbf {a}}(0)$ in the Eq. (8) represents expected present values at $t=0$ of *an irredeemable mortgage annuity* paid by each obligor according to its rating grade.

In order to consider the possibility to recover part of the credit if an obligor defaults, we should adjust the value of ${\mathbf {a}}(0)$ accordingly. To this end, Eq. (8) should be modified to take this effect into account. Introducing the loss-given-default (LGD), $(1-rr)$, and letting *rr* be the recovery rate,^18^ the vector of *expected values of the recovery rate by credit risk class* for $t\rightarrow \infty$ is^19^:

9 $$\begin{aligned} {\mathbf {r}}(0)=rrQ\left[ (I-\Lambda ^{-1})B^{\prime }\right] Q^{-1}{\mathbf {v}}. \end{aligned}$$

Following a unitary-payment perpetual amortizing scheme and allowing for partial recovery of funds in case of default, the present value of an *expected positive exposure* ${\tilde{a}}_{j}$ must always satisfy the equivalence ${\tilde{a}}_{j}(1-r_{j})=a_{j}$, where $r_{j}<1$ is *j*-th element of the vector ${\mathbf {r}}_{0}$. Bearing this in mind, the final expectation of a *unitary perpetual annuity value* at time $t=0$ calculated for each obligor according to its rating grade *j* is then:

10 $$\begin{aligned} {\tilde{a}}_{j}=\frac{a_{j}}{1-r_{j}}. \end{aligned}$$

The vector ${\tilde{\mathbf {a}}}(0)$, whose elements are the values $\tilde{a}_{j}$, can be interpreted as a set of perpetual annuities based on *fundamental risk metrics* inherent to obligors labelled with specific credit risk class.

### A.3 Portfolio pricing

Equation (10) allows to establish conditions for *credit portfolio pricing* and *Debt Agency financial equilibrium*, once the functional form for the portfolio default probability $cdp_{_{W}}(t)$ for $t\ge 0$ has been specified.

#### **Proposition 5**

*Given the initial portfolio asset allocation* ${\mathbf {w}}_{0}^{T}=\left[ w_{1},\ldots ,w_{j},\ldots w_{n}\right]$, *with* $w_{j}=d_{j}/TD$, *the Debt Agency will price the risks for* $t\ge 0$ *using the portfolio expected default probabilities* $E(cdp_{_{W}}(t))={\mathbf {w}}_{0}^{T}Q\Lambda ^{t}Q^{-1}v$ *and setting the overall annual payments* $I_{W}(t)$ *at time* $t=0$ *so that its financial equilibrium holds in expectations*:

11 $$\begin{aligned} TD=I_{W}(0){\tilde{a}}_{W}(0)=I_{W}(0){\mathbf {w}}_{0}^{T}{\tilde{\mathbf {a}} }(0)=I_{W}(0)\sum _{j=1,\ldots ,n}w_{j}{\tilde{a}}_{j}. \end{aligned}$$

#### *Proof*

In analogy with formula (10), let be ${\tilde{a}}_{W}(0)$ such that ${\tilde{a}}_{W}(0)(1-r_{W}(0))=\sum _{j=1,\ldots ,n} w_{j}a_{j}$, with $r_{W}(0)=\sum _{j=1,\ldots ,n}z_{j}r_{j}$ and $\sum _{j=1,\ldots ,n}z_{j}=1$, we have:

$$\begin{aligned} {\tilde{a}}_{W}(0)\left( \sum _{j=1,\ldots ,n}z_{j}-\sum _{j=1,\ldots ,n}z_{j}r_{j}\right)&=\sum _{j=1,\ldots ,n}w_{j}a_{j}\\ \sum _{j=1,\ldots ,n}z_{j}{\tilde{a}}_{W}(0)(1-r_{j})&=\sum _{j=1,\ldots ,n}w_{j}a_{j}. \end{aligned}$$

A non trivial solution of this equation is $z_{j}{\tilde{a}}_{W}(0)=w_{j} \frac{a_{j}}{(1-r_{j})}=w_{j}{\tilde{a}}_{j}$, for $j=1,\ldots ,n$. Summing up for *j*, we obtain ${\tilde{a}}_{W}(0)\sum _{j=1,\ldots ,n}z_{j}=\sum _{j=1,\ldots ,n} w_{j}{\tilde{a}}_{j}$, i.e. ${\tilde{a}}_{W}(0)=\sum _{j=1,\ldots ,n}w_{j}{\tilde{a}} _{j}$. It also follows that $z_{j}$ is a linear combination such that:

$$\begin{aligned} z_{j}=\frac{w_{j}{\tilde{a}}_{j}}{\sum _{j=1,\ldots ,n}w_{j}{\tilde{a}}_{j}}. \end{aligned}$$

$$\square $$

However, the solution $I_{W}(0)$ is not the only one solving the Eq. (11). In fact, letting the vector ${\mathbf {c}}_{t}^{T}=\left[ c_{1},c_{2},\ldots ,c_{j},\ldots c_{n}\right]$ represent the overall payments due at time $t=0$ by each credit risk class *j*, the financial equilibrium requires that the sum of their expected value, ${\mathbf {c}}^{T}{\mathbf {a}}(0)$, and the overall expected recovery value, ${\mathbf {d}}^{T}{\mathbf {r}}(0)$, be equal to the total credit portfolio holding *TD*:

12 $$\begin{aligned} TD=\sum _{j=1,\ldots ,n}d_{j}={\mathbf {c}}^{T}{\mathbf {a}}(0)+{\mathbf {d}}^{T} {\mathbf {r}}(0)=\sum _{j=1,\ldots ,n}c_{j}a_{j}+{\mathbf {d}}^{T}{\mathbf {r}}(0). \end{aligned}$$

The solution value for $c_{j}$ (12) can be found for infinite arbitrary combinations of the other $c_{i}$, $i\ne j$. General financial criteria of course apply to determine “admissible” values to the $c_{j}.$ A special solution, that we call here *idiomatic fundamental pricing solution*, consists in relating the payments of the *j*-th class to the corresponding debt level and riskiness:

13 $$\begin{aligned} c_{fj}=\frac{d_{j}(1-r_{j})}{a_{j}}=\frac{d_{j}}{{\tilde{a}}_{j}}. \end{aligned}$$

The specific feature of this solution is that each obligor *pays for the risk inherent to the specific credit risk class to which it is assigned, without any form of solidarity or mutuality among obligors of different classes.* Nevertheless, the solution (13) does not price in line with portfolio expected default probabilities, giving rise to the following straightforward proposition.

#### **Proposition 6**

*Be* $I_{F}(0)=\sum _{j=1,\ldots ,n}c_{fj}=\sum _{j=1,\ldots ,n}\frac{d_{j}}{{\tilde{a}} _{j}}$, *if we price the portfolio* ${\mathbf {d}}^{T}$ *using portfolio expected default probabilities*, $E(cdp_{W}(t))={\mathbf {w}}_{0}^{T}Q\Lambda ^{t} Q^{-1}{\mathbf {v}}$ for $t>0$, *the total payments at* $t=0$ *are such that* $I_{F}(0)\ge I_{W}(0)$.^20^

#### **Proposition 7**

*If for* $t=0$ *the Debt Agency computes the fair value of the portfolio* *pv*(0) *using portfolio default probabilities but charges obligors individually by using the idiomatic* fundamental pricing *formula* (13), *so that* $pv(0)=I_{F}(0){\tilde{a}}_{W}(0)$, *then*:

- *the portfolio fair value will be greater than the* *TD*, *thus generating for the agency a positive economic value of equity* $eve(0)=pv(0)-TD$
  $$\begin{aligned} pv(0)\ge TD \end{aligned}$$
- *the economic value of equity can be remunerated at a positive interest rate*:
  $$\begin{aligned} fc\le \left[ (I_{F}(0)-I_{W}(0))/eve_{0}\right] . \end{aligned}$$

#### *Proof*

From Eq. (11) it follows that

$$\begin{aligned} pv(0)=I_{F}(0){\mathbf {w}}_{0}^{T}{\tilde{\mathbf {a}}}_{{\mathbf {0}}}\ge I_{W}(0){\mathbf {w}}_{0}^{T}{\tilde{\mathbf {a}}}_{0}=TD. \end{aligned}$$

$$\square $$

#### *Remark* 8

The total payment $I_{W}(0)$ obtained under the equilibrium condition (11) is structurally lower than the amount due in the idiomatic fundamental pricing configuration. This is attributable to a “pooling effect”, i.e., in our case, to the fact that, within a portfolio approach, the transition probabilities among credit risk classes entail a risk mitigation benefit, since they imply, in each year of observation, potential improvements for the worst credit risk classes.

The Proposition 5 is relevant because it shows that the Debt Agency generates value for potential investors, collecting additional risk capital that can be used for solvency purposes. Also relevant is the question of overall cost distribution among obligors. Here we only want to emphasise that the overall cost of the portfolio obviously depends on the cost distribution rule that we adopt to charge each risk class.

#### **Proposition 9**

*Under the pricing rule defined by* (11), *if we charge each credit risk class* *j* *in proportion to its debt*, *i.e. using the weight* $wj=dj/TD$, *then we equalize the price of risk uniformly*, *thus ending up mutualizing part of the debts among classes*.

#### *Proof*

Under (11) set the portfolio unit cost of the risk equal $ucr_{_{W}}=I_{W}(0)/TD$. Now consider the idiomatic fundamental risk pricing rule under (13) and set the unit cost of the risk for the credit risk class *j* equal $ucr_{j}=$ $c_{fj}/d_{j}$. For an obligor *i* of credit risk class *j* and nominal debt $\delta _{ji}$, if $ucr_{j} >ucr_{_{W}}$ then its equivalent nominal debt $\delta _{ji}^{\prime }$ is

$$\begin{aligned} \delta _{ji}^{\prime }<\frac{ucr_{_{W}}}{ucr_{j}}\delta _{ji}. \end{aligned}$$

$$\square $$

From Proposition 5, the excess cost of $\left( I_{F}(0)-I_{W}(0)\right)$ $\ge 0$ could possibly be allocated among credit risk classes without compromising the agency’s financial equilibrium.

### A.4 Intertemporal equilibrium

Thus far, we have supposed that the Debt Agency (1) determines its periodic cash flow according to an irredeemable amortization scheme; (2) prices risks using the metrics (5); and that (3) no extra provisions or capital charges for unexpected losses or other risks are needed (in the Appendix on solvency capital we will challenge and supersede this assumption). Since at time $t\ge 0$ the proposition (4) always holds true, we illustrate the financial equilibrium of the Debt Agency by using the pricing formula given by the Eq. (11) and we leverage on a *fundamental characterization of our agency institutional framework*, which will be fully explained in the Appendix on solvency capital. Given the agency asset allocation, ${\mathbf {w}}_{t}^{T}=\left[ w_{1},\ldots ,w_{j},\ldots w_{n}\right]$ , and the portfolio intertemporal default probability $cdp_{_{W}}(t)$ up to time *t*, we characterize the proportion of defaults at portfolio level, $w_{n}$, as represented by the following process:

14 $$\begin{aligned} w_{n}=E[cdp_{_{W}}(t)]={\mathbf {w}}_{t-1}^{T}Q\Lambda Q^{-1}{\mathbf {v}} \end{aligned}$$

with $\sum _{j=1,\ldots n}w_{j}=1$ and ${\mathbf {v}}$ a null vector apart from its last element equal to 1.

#### *Remark* 10

The formula (14) states that, although the asset allocation can evolve considering *erratic rating grade migration* and that the non-default classes can diverge considerably from their expected value, on the contrary, the default class always must evolve according to its expected value. In the Appendix on solvency capital, we will show that this setting is consistent with the particular meaning of default that works effectively for Member States in the institutional context of the Eurozone. This device allows to cumulate the share of debtors that have “theoretically” defaulted at every time *t*, which is necessary to correctly calculate the agency intertemporal equilibrium. Note that future portfolio default probabilities will be the result of actual rating grade migrations.

In developing the agency intertemporal equilibrium, we resort to the following statements:

1. the appropriate average loss-given-default (LGD) rate is $(1-rr_{_{W}} )$, with $rr_{_{W}}$ representing percentage of nominal debt recovered in the case of default (i.e. the portfolio recovery rate)
2. the agency’s annual discount rate used to compute present values is $\pi _{t}$
3. the agency’s reserves deposit is remunerated at an annualized interest rate $\pi _{t}^{\prime }$ set by the Central Bank, which is conceived as the long term equilibrium rate of its monetary policy
4. the agency’s liability equals $TD=\sum _{j}d_{j}$ and it is rolled over for an infinite span of time, issuing and renewing at pair indexed bonds of unitary maturity (e.g. 1 year) of overall face value equal *TD*
5. the agency’s funding cost $FC=\pi _{t}^{\prime \prime }TD$ is determined using an annual interest rate of $\pi _{t}^{\prime \prime }$.

If we suppose that the Debt Agency can reprice obligors’ funding costs by using the Eq. (11), then its net exposure at time *t* is subject to the following constraint, which has to be solved for $I_{W}(t)$:

15 $$\begin{aligned} TD-rs(t)=pv_{W}(t)=I_{W}(t){\tilde{a}}_{W}(t)=I_{W}(t){\mathbf {w}}_{t} ^{T}{\tilde{\mathbf {a}}}_{t} \end{aligned}$$

where $pv_{W}(t)$ is the present value of future cash flow and *rs*(*t*) is the total cumulated reserve deposit at the Central Bank, given by:

16 $$\begin{aligned} rs(t)&=rs(t-1)(1+\pi ^{\prime })+I_{W}(t-1)[1-(cdp_{_{W}}(t)-cdp_{_{W} }(t-1))]+ \\ &\quad +rr_{_{W}}TD(cdp_{_{W}}(t)-cdp_{_{W}}(t-1))-FC \end{aligned}$$

with $rs(0)=0.$ Given the portfolio share of defaulted obligors $(cdp_{_{W} }(t)-cdp_{_{W}}(t-1))$ in the interval $[t-1,t]$ , $rr_{_{W}}TD(cdp_{_{W} }(t)-cdp_{_{W}}(t-1))$ represents the cash inflow due to recovery from defaulted obligors. Note that *rs*(*t*) is a stochastic realization of the process $cdp_{W}(\tau )$ for $\tau$ $\le t$, which is known at time *t*. This equilibrium implies that in the case of adverse risk migrations in the interval $[t-1;t]$, the surviving obligors will bear a greater cost for $I_{W}(t)$ in order to assure the agency equilibrium over time, according to (15).

Considering the three mentioned rates $$\pi _{t},\pi _{t}^{\prime }\ $$and $\pi _{t}^{\prime \prime },$ to be noted is that:

1. they are expectations conditional on information available at time *t*;
2. by institutional design, the agency will fix the $\pi _{t}$ rate equal to the Central Bank’s long-term rate $\pi _{t}^{\prime }$;
3. by design, also the agency issuances are indexed to $\pi _{t}^{\prime }$;
4. the rate $\pi _{t}^{\prime \prime }$ will always be such that $\pi _{t}^{\prime \prime }\le$ $\pi _{t}^{\prime }$, since we make the assumption that the Central Bank can always buy the residual issuance in order to ensure the alignment of the yield to $\pi _{t}^{\prime }$ (see the institutional role to respect to the Debt Agency assigned to the ECB in Sect. 3).

#### **Proposition 11**

*For* $t\ge 0$, *if* $\pi _{t}=\pi _{t}^{\prime }=\pi _{t}^{\prime \prime }$ *and the agency reprices the obligors cost at every* *t* *using Eq.* (11), *then* $TD=pv_{W}(t)+rs(t)$: *the agency balance sheet asset-side always equates the liability-side*, *and the agency equilibrium is assured over time*.

#### *Proof*

The proposition follows directly from Eq. (15), which states that for the portfolio fair value at time *t* we have $pv_{W} (t)=I_{W}(t){\tilde{a}}_{W}(t)$ , then:

$$\begin{aligned} pv_{_{W}}(t)+rs(t)=TD \end{aligned}$$

with $$pv_{W}(0)=TD.$$ $$\square $$

#### *Remark* 12

Note that, for $t\longrightarrow \infty$ , since $pv_{_{W}}(t)\longrightarrow 0$ then $rs(t)\longrightarrow TD$ and the agency will have piled up enough reserves to repay the nominal value of its bonds.

#### **Proposition 13**

*For* $t\ge 1$, *if* $\pi _{t}=\pi _{t}^{\prime }=\pi _{t}^{\prime \prime }$ *then returns from the Debt Agency’s asset side are* expected *to remunerate the liability side*, *i.e.* $(pv_{W}(t)+rs(t))(1+\pi )=TD+FC.$^21^

We have shown that, since the Debt Agency is able to align:

1. interest rates used to compute revenues and present values
2. returns on reserves in form of deposits at the Central Bank
3. the cost of funding,

then, by the Propositions 6 and 7 the Debt Agency will always be able, in expectation, to back liabilities with the fair value of its assets. This entails that, in an “arm’s length transaction”, as prescribed by standards such as Solvency II and Basel III, the agency will always be able to repay its overall debt of *TD* whenever requested.

### A.5 Solvency capital

So far, we have considered that the Debt Agency prices its own risks using the expected default probabilities term-structure with infinite granularity of obligors in each credit risk class of the underlying portfolio.

Within a “closed portfolio” irredeemable framework, default outcomes deal only with the “when” of their occurrence, since the portfolio cumulative default probability over an infinite time span always equals 1, given that the TTC matrix is recursive and has one absorbing state, coinciding with the default state. As a consequence, when the default-term-structure evolves, the pricing operated by the Debt Agency will allow it to accumulate enough reserves or adjust pricing to maintain its financial equilibrium according to Eq. (15).

What if, however, the events of default anticipate and are much more concentrated? Such an eventuality would violate the assumed hypothesis of infinite granularity of obligors, thus causing the Debt Agency to remain with insufficient accumulated reserves and with a lack of revenues to cover its future expected liabilities.

First of all, we should speculate what would be a default of a Member State once the Debt Agency has been established. Should a Member State incur a state of insolvency, it is nonetheless likely that, under a suitable “ex-ante budget control regime” necessary to assure the agency‘s ongoing correct operational course, the failing Member State will soon or later be able to restore its ability to pay its future instalments. This means that the Member State would undergo a period of restructuring before full recovery and that, in reality, the default of a Eurozone Member State should be considered more properly a state of “*forbearance*”. Moreover, to be noted is that, during this finite period, the distressed asset corresponding to Member State‘s debt does not need to be written off but will remain “frozen” on the Debt Agency balance sheet. Consequently, because of the reduced ability of the Member State to pay its overdue instalments, the Debt Agency will not be able to maintain its equilibrium and to finance its funding costs.

However, if adequately complemented with a suitable insurance scheme, this “restructuring nature” of a failing Member State allows us to maintain some essential features of our model, even outside our hypothesis of infinite granularity of obligors. We are then entitled to transform the initial hypothesis into an equivalent one, according to which the *debt of each Member State is assumed to be infinitely divisible into infinitesimal parts*, supposed mutually independent only for mathematical convenience. This methodological assumption enables us to proceed in our calculations as if only a portion of a Member State s debt was recorded as a loss, according to the expected default probability of the rating grade class of the Member State in the interval $(t;t+1)$. As a consequence, the portfolio default probability is also assumed to follow the process under (14).

Thanks to this “fiction”, the cost of debt would be re-estimated in each period only on the basis of the following risk factors:

1. the migration risk, when the agency asset allocation by credit risk classes is changed due to effective Member State risk up-grade or down-grade, and
2. changes in the expected monetary policy rates of the Central Bank.

On this line of reasoning, the repricing mechanism of the Eq. (15) will ensure that the pricing applied by the agency allows the maintenance of its financial equilibrium. Moreover, and in order to fully implement that setting, we need:

1. to adjust the annual pricing to take account of the theoretical loss as mentioned,
2. to allow for an insurance scheme designed to provide financial support in the form of a capital equivalent to the present values of annual payments lost during the forbearance period of a Member State.

As regards the first point, our fiction consists in imagining that a single Member State *i* with initial debt equal $D_{ij}$, representing a significant fraction of a specific credit risk class *j*, will pay only an amount determined using the partition rule under (13) adjusted in proportion to the theoretical default for each given interval $[t,t+1]$ as follows:

$$\begin{aligned} {\tilde{c}}_{ij}(t)=\left[ {\tilde{c}}_{ij}(t-1)-rec_{ij}(t-1)\right] (1-E\left[ cdp_{j}(t)-cdp_{j}(t-1)\right] )+rec_{ij}(t) \end{aligned}$$

where ${\tilde{c}}_{ij}(0)=\frac{D_{ij} }{{\tilde{a}}_{j}}$ according to formula (13), $\tilde{a}_{j}$ and $cdp_{j}(t)$ are credit risk class characteristic quantities the meaning of which we have widely discussed in the previous sections, and $rec_{ij}(t)$ the recovery proportion such that:

$$\begin{aligned} rec_{ij}(t)=rr_{_{W}}D_{ij}E\left[ cdp_{j}(t)-cdp_{j}(t-1)\right] \end{aligned}$$

with $rr_{_{W}}$ the theoretical portfolio recovery rate. Note that $\lim _{t\rightarrow \infty }$ ${\tilde{c}}_{ij}(t)=0$, in line with the amortizing nature of the plan developed within this framework.

As for the second point, the Debt Agency will only need to relay upon an available capital endowment to cover the *temporary shortage of cash inflow* due by an Member State in state of forbearance. This capital allowance, which takes here the form of an insurance, should then be proportional to the total annual cash flows at risk for a period of time, *fi*, that we call forbearance interval. The present value at time *t* of future payments due during the forbearance in the interval $[t,t+fi]$ is:

$$\begin{aligned} fp_{ij}(t)= {\textstyle \sum \limits _{h=t}^{t+fi}} \frac{{\tilde{c}}_{ij}(h)}{(1+\pi )^{h-t}}. \end{aligned}$$

The periodic premium that should be paid is then given by:

17 $$\begin{aligned} prem_{ij}(t)=\frac{ {\textstyle \sum \limits _{\tau =t}^{+\infty }} \left( \frac{fp_{ij}(\tau )}{(1+\pi )^{t}}\right) (cdp_{stress,j} (\tau +1)-cdp_{stress,j}(\tau ))-rm_{i}(t)}{a_{j}} \end{aligned}$$

where $a_{j}$ is the *j*-th element of the vector ${\mathbf {a}}(t)$, $\pi$ is a suitable discounting rate, and the $cdp_{_{stress,j}}$ represents a stressed default probability obtained through the Eq. (4) by using a suitable confidence interval such that:

$$\begin{aligned} P_{cdp_{w}}(x\le cdp_{stress})=\alpha \end{aligned}$$

with $\alpha$ a prudential probability threshold. The $rm_{i}(t)$ represents the cumulated mathematical reserve at time *t* given by:

$$\begin{aligned} rm_{i}(t)=rm_{i}(t-1)(1+\pi )+prem_{ij}(t)-{\varvec{1}}_{[fi]} (t)fp_{ij}(t) \end{aligned}$$

where ${\varvec{1}}_{[fi]}(t)$ is equal to one when *t* is the first year of a forbearance period. Note that this reserve is different from the reserve deposit *rs*(*t*). To include the insurance premium, a suitable mark up should be considered by adjusting overall payments accordingly. The final cost for the Member State *i* of rating grade *j* is then equal to:

$$\begin{aligned} {\hat{c}}_{ij}(t)={\tilde{c}}_{ij}(t)+prem_{ij}(t). \end{aligned}$$

If we define ${\hat{I}}_{F}(t)=\sum _{j=1,\ldots ,n}[{\tilde{c}}_{ij}(t)+prem_{ij}(t)]$ then the following inequalities hold:

$$\begin{aligned} {\hat{I}}_{F}(t)\ge I_{F}(t)\ge I_{W}(i). \end{aligned}$$

This shows that the economic equilibrium of the Debt Agency is assured.

#### *Remark* 14

Following Proposition 5, we have shown that under the pricing configuration (13) the agency has the potential to generate a *positive economic value of equity,* which in our numerical elaborations turns out to be of the same order of magnitude as the present value of future insurance premiums, partly mitigating the costs required to finance the aforementioned insurance scheme.

Another important source of risk for the agency is interest rate volatility. We argue that the agency architecture hereby proposed can be thought of as a shield to protect Member States against “*liquidity spread risk*”, thanks to the link with the Central Bank and the insurance scheme to cope with unexpected sovereign default risk. Nonetheless, interest rate asymmetric movements can cause repricing risk. We supposed that the agency will roll over its debt by issuing bonds indexed to prevailing Central Bank fund rates, but there is always the possibility that alignment will fail to be effective. In this case the agency will always have the ability to reprice the total instalment needed to restore its financial equilibrium, by applying the equilibrium formula under Eq. (15).

### A.6 Rebalancing of the instalments

#### *Proof*

Define the Lagrangian:

$$\begin{aligned} L(w_{1}^{\prime },\ldots ,w_{j}^{\prime },\ldots w_{n}^{\prime };\lambda )=\sum _{j=1,\ldots ,n}I_{W}(t)^{2}{\tilde{a}}_{j}^{2}\left( w_{j}^{\prime }-\frac{c_{f,j} }{I_{W}(0)}\right) ^{2}-\lambda \left( \sum _{j=1,\ldots ,n}w_{j}^{\prime }-1\right) . \end{aligned}$$

First order conditions give:

$$\begin{aligned} \frac{\partial L}{\partial w_{j}^{\prime }}&=2I_{W}(t)^{2}{\tilde{a}}_{j} ^{2}(w_{j}^{\prime }-\frac{c_{f,j}}{I_{W}(t)})-\lambda =0\\&=w_{j}^{\prime }-\frac{c_{f,j}}{I_{W}(t)}-\frac{\lambda }{2I_{W}(t)^{2} {\tilde{a}}_{j}^{2}}=w_{j}^{\prime }-\frac{c_{f,j}}{I_{W}(t)}-\lambda ^{\prime }{\tilde{\pi }}_{j}^{2}\text{, with }\lambda ^{\prime }=\frac{\lambda }{2I_{W}(t)^{2}}\\ \frac{\partial L}{\partial \lambda }&=\sum _{j=1,\ldots ,n}w_{j}^{\prime }-1=0. \end{aligned}$$

Summing up by *j* and considering the constraint, we have:

$$\begin{aligned} \lambda ^{\prime }\sum _{j=1,\ldots ,n}{\tilde{\pi }}_{j}^{2}&=\sum _{j=1,\ldots ,n} w_{j}^{\prime }-\frac{I_{F}(t)}{I_{W}(t)}=\frac{I_{W}(t)-I_{F}(t)}{I_{W}(t)}\\ w_{j}^{\prime }I_{W}(t)&=c_{f,j}-\left( I_{F}(t)-I_{W}(t)\right) \frac{{\tilde{\pi }}_{j}^{2}}{\sum _{j=1,\ldots ,n}{\tilde{\pi }}_{j}^{2}}. \end{aligned}$$

$$\square $$

## Footnotes

1 “It also announced three additional TLTRO III operations to be conducted between June and December 2021 and raised the total amount that banks are entitled to borrow in these operations. The ECB also plans to conduct four additional pandemic emergency longer-term refinancing operations (PELTROs) in 2021.” ECB (2021).

2 In this paper we consider only the proposals that aim to jointly solve the problem of the scarcity of safe assets and the financeability of public debts in Europe. Another approach is to build a safe asset without worrying about public debt financing (Salto et al. 2020).

3 With the term ‘liquidity risk’ we refer to the effects caused by the price dynamics (secondary markets) on public bonds refinancing and repricing by Member States (primary markets).

4 The current cost paid by each state is thus filtered by its cost of liquidity and the ‘global market sentiment’ (investors’ risk aversion).

5 Here, we refer mainly to the controversy involving the German Supreme Court—Bundesverfassungsgericht—and the Court of Justice of the European Union).

6 This does not imply nor exclude a role of the central bank as lender of last resort. The DA is neutral with respect to both options.

7 For a demonstration of this proposition, see the previous version of this work, available at https://papers.ssrn.com/sol3/papers.cfm?abstract_id=3579496. For any further information on the analytics, please write to massimo.amato@unibocconi.it

8 Standard and Poor’s Sovereigns Ratings have been downloaded from Bloomberg using a query with parameters: - RTG_SP_LT_LC_ISSUER_CREDIT - RATING_AS_OF_DATE_OVERRIDE - Sovereign Issuer Ticker.

9 It could be objected that the statistics relating to the default of sovereign debt reported in the transition matrix are not reliable, since the events are so rare that they can cause problems of robustness. This is in general a very well founded objection, but it does not concern the transition frequencies between rating grades, which are instead our main source of information with which to derive a reliable measure of the cost of risk associated with each sovereign debt. In fact, as our analyses and verifications show, there is a strong correlation between rating grade transition frequencies and credit cycle.

10 Source: ECB 2021.

11 This iterative procedure was carried out as follows: once determined, with weights equal to sovereign debts, the average portfolio yield at the level of the Euro Area, *M*(*Y*(*t*)), $\pi _{0}$ was set equal to *M*(*Y*(*t*)) and the idiomatic cost $IP_{t}(\pi _{0})$, associated with the same portfolio, was calculated. We then proceeded to the subsequent iteration *i* by positing

$$\begin{aligned} \pi _{i}=E(Y(t)-\alpha \cdot [E(Y(t)-IP_{t}(\pi _{i-1})] \end{aligned}$$

with $\alpha$ representing an appropriate sensitivity parameter. The procedure ended when

$$\begin{aligned} (\pi _{i}-\pi _{i-1})<\varepsilon \end{aligned}$$

with $\alpha =1$. In this way, a proxy was obtained that could be considered an appropriate discount rate to be used for determining the discount rate as illustrated in the Appendix A.2. We repeated this procedure for each given time step in the yield time series.

12 Source: ECB 2021.

13 Source: Refinitiv 2021.
